# The impact of confidentiality and privacy concerns on behavioral intention to adopt electronic personal health records among chronic patients in southwest Ethiopia in 2023: an application of the UTAUT2 model

**DOI:** 10.3389/fdgth.2025.1475460

**Published:** 2025-10-31

**Authors:** Sisay Yitayih Kassie, Adamu Ambachew Shibabaw, Alex Ayenew Chereka, Abiy Tasew Dubale, Addisalem Workie Demsash, Yitayish Damtie, Habtamu Setegn Ngusie, Agmasie Damtew Walle

**Affiliations:** ^1^ Department of Health Informatics, College of Health Science, Mettu University, Mettu, Ethiopia; ^2^ Department of Public Health, College of Medicine and Health Science, Injibara University, Injibara, Ethiopia; ^3^ Department of Health Informatics, School of Public Health, College of Medicine and Health Sciences, Woldia University, Woldia, Ethiopia

**Keywords:** personal health record, chronic patients, adoption, behavioral intention, UTAUT2

## Abstract

**Background:**

The use of electronic personal health record systems (e-PHRS) is essential for chronic patients as they help to improve self-care management and communication between caregivers. However, when implementing e-PHRS, patients often express their concerns regarding privacy and confidentiality issues. Therefore, this study aimed to assess the impact of confidentiality and privacy concerns on the level of intention to use e-PHRS among chronic patients in southwest Ethiopia in 2023.

**Method:**

A cross-sectional study was conducted among 680 chronic patients from 24 July to 17 September 17 2023 in southwest Ethiopia. A translated interviewer-administered questionnaire was used to collect the data. A systematic random sampling approach was employed to recruit the study participants. The impact of confidentiality and privacy concerns on the patients' intentions regarding e-PHRS adoption was examined using the extended Unified Theory of Acceptance and Use of Technology 2 (UTAUT2) model. We used measurement and structural model statistics to assess the validity of the proposed model. All the hypotheses were tested using structural equation modeling and presented using SPSS AMOS version 23. Standardized regression coefficients (*β*), 95% CIs, and *p*-values < 0.05, indicating significance, were used to examine the association between the exogenous and endogenous variables.

**Result:**

A total of 680 chronic patients, with a response rate of 87.3%, were included in the study. In total, 288 participants (42.4%) intended to adopt e-PHRS (95% CI: 39.0, 46.2). The results show that the extended UTAUT2 model explained approximately 75% of the variance in e-PHRS adoption. Confidentiality (*β* = 0.156, *p* < 0.01), privacy (*β* = 0.216, *p* < 0.05), and social influence (*β* = 0.157, *p* < 0.01) were significantly associated with adopting e-PHRS. Social influence and facilitating conditions were found to mediate confidentiality and behavioral intention, with a *p*-value < 0.001, while only social influence mediated the privacy concern and behavioral intention to adopt e-PHRS, with a *p*-value < 0.001.

**Conclusion:**

Less than half of the surveyed patients intended to use e-PHRS. The result confirmed the significant impact of confidentiality and privacy concerns on e-PHRS adoption. As a result, this study demonstrates that confidentiality and privacy concerns are two main challenges that stakeholders and program developers should consider during the implementation of e-PHRS in low-income countries.

## Introduction

Since the beginning of the twenty-first century, healthcare services have undergone significant changes (1, 2). Health service delivery has changed from a doctor-knows-best model to one where individuals are encouraged to take an active role in their health and make inclusive healthcare decisions (3). With the advancement of health information technology, patients have been encouraged to take more responsibility for their health and wellbeing.

The World Health Organization (WHO) is increasingly recognizing the benefits of information technologies for patients’ wellbeing. For instance, electronic personal health records (e-PHRs) are considered an alternative to empowering patients' self-care management practices (4). An e-PHRS is an application that allows individuals to access, share, track, maintain, and manage their health, and acts as a communication channel. It also increases patient involvement in making decisions about their health condition and sharing their health information (5, 6). By using e-PHRS, patients can improve their self-efficacy and self-care management practice (7–9). Patients who use e-PHRS can play an active role in their health and can directly gather, store, and access a wide array of credible health information (10).

Due to this, healthcare organizations adopt e-PHRs to achieve three goals: healthcare access, a reduction in costs related to visiting the healthcare organization, and improved quality of healthcare delivery. They also help empower patients and provide continuity of patient care and the patient-provider partnership, individual control, and engagement in decision-making (10–12). In addition, e-PHRs provide numerous benefits for healthcare professionals in retrieving patient information, accessing and modifying patient data, sending automated reminders to prevent medication errors, improving the health information exchange between providers and patients, and ensuring thorough and clear documentation of patients’ health and clinical conditions (13). A European study showed that e-PHRs significantly changed how patients receive medical care (14–16). Another study conducted in Portugal found that e-PHRs help patients manage their health and take an active role in their care (10, 17, 18). Furthermore, a study conducted in the United Kingdom revealed that e-PHRs allow patients to communicate with a doctor, make repeat prescription requests, and schedule appointments (19). In addition, a study conducted in Australia found that e-PHRs enhance patient participation in decision-making, information sharing, and self-management, and increase patient empowerment (20). In developing countries, governments have encouraged individuals to use e-PHRs. However, e-PHRs are not widely used. For example, a study conducted in Ethiopia among chronic patients showed that 46.7% of them intended to use e-PHRs to manage their health (21). Another study conducted in Ethiopia revealed that 57.6% of healthcare professionals used e-PHRs (22, 23). Several studies have recognized a lack of training, knowledge, and attitudes as a challenge and barrier to the use of e-PHRs (24, 25). However, most importantly, the researchers identified that the primary problem is patients' data confidentiality and privacy concerns (26–28). Evidence shows that 54% and 59% of respondents were concerned about their data when using e-PHRs. The problem has increased significantly since the platform is not developed to identify sensitive information from non-sensitive information. These become more pressing when patients are required to upload sensitive and personal health information to online platforms (29).

As a result, evidence suggests that the adoption rate of e-PHRs in developing countries such as Ethiopia is inadequate. The challenges to adopting e-PHRs extend beyond privacy and confidentiality concerns; they also include social influences, perceived ease of use, perceived usefulness, and self-efficacy. Among these, privacy and confidentiality issues remain the primary obstacles to the acceptance of these new systems (26). Privacy and confidentiality concerns exacerbate patients’ reluctance to adopt e-PHRs (26, 29). This is supported by a study conducted among American customers that indicated that 55% of them were not interested in using e-PHRs due to their personal data confidentiality and privacy concerns (30). In addition, numerous factors play a significant role in adopting and using information technologies (31). This underpins the importance of gaining a deeper understanding of various barriers in the implementation of e-PHRs through the lens of the current Unified Theory of Acceptance and Use of Technology version 2 (UTAUT2) model (32). Therefore, this study aimed to examine the impact of confidentiality and privacy concerns on chronic patients’ behavioral intentions to adopt e-PHRs in southwest Ethiopia in 2023 using the UTAUT2 model. A deep understanding of the impact of confidentiality and privacy on behavioral intention to adopt e-PHRs is a crucial step in setting strategies and policies. The results of this study will help strategy consultants, policy developers, policymakers, and ministers of health to make evidence-based decisions on the adoption of e-PHRs.

### Theoretical background and hypothesis

Several notable models have been introduced to investigate factors that influence the adoption of health information technology. From 2003 to 2012, Venkatesh's research developed the UTAUT, which provides a comprehensive conceptual framework to explain information technology's adoption, acceptance, and intention. However, the UTAUT2 model is the most well-known and used model based on recent research findings to explain the end-users' technological acceptance and use (33–35). It has been selected for several reasons, including its success in assessing factors that affect patients' behavioral intention, with a high explanatory power (36–39). In addition, the UTAUT2 includes a comprehensive core model that enables researchers to improve the model further by adding external factors, such as confidentiality and privacy concerns, to measure their effect on behavioral intention to adopt health information technology.

**Figure 1:**
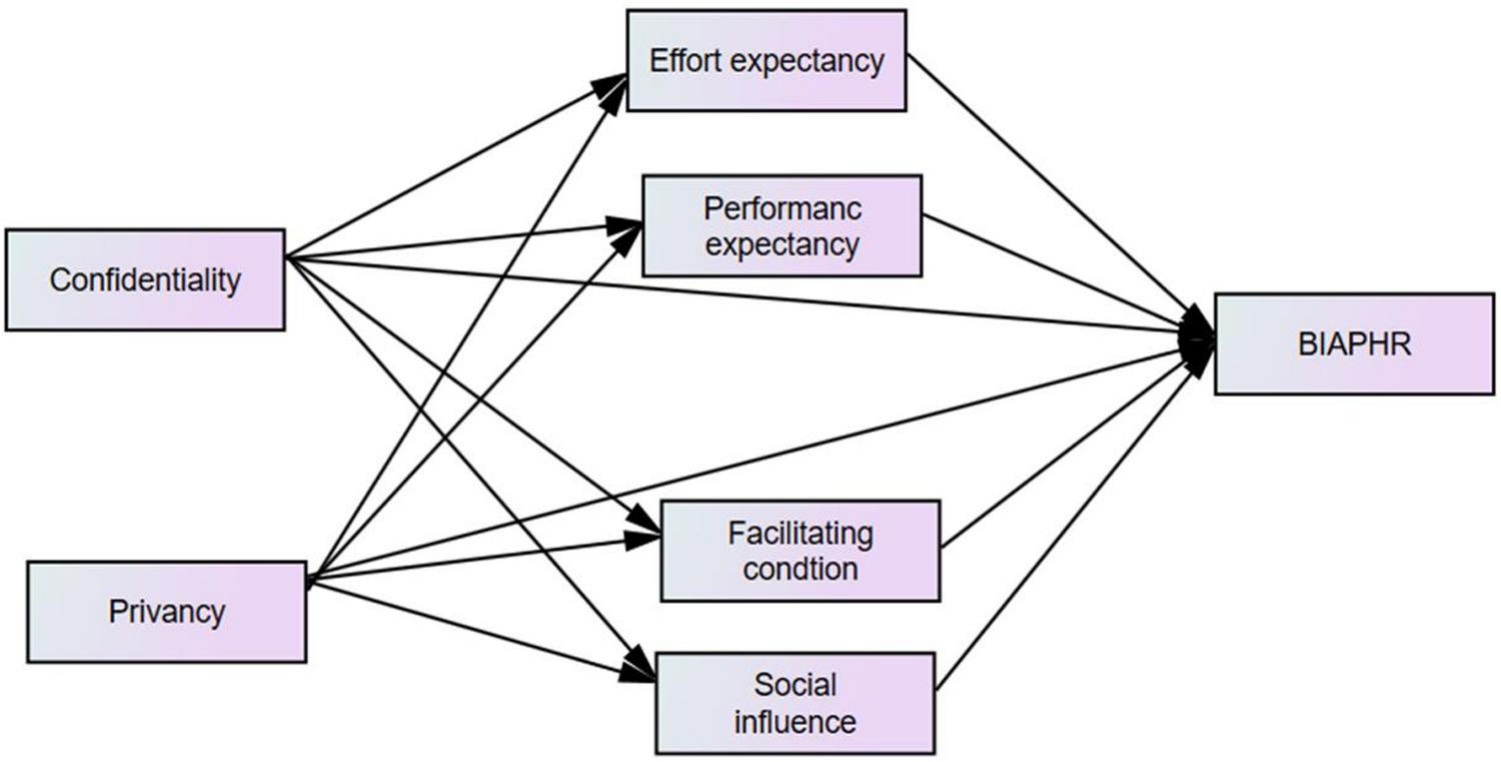
Conceptual framework of the adapted UTAUT2 model for this study on the behavioral intention to adopt e-PHRs among chronic patients and its predictors in southwest Ethiopia in 2023.

As a result, using the appropriate theory or model as a theoretical basis to best explain users' behavioral intention to adopt e-PHRs is critical in answering the research questions and investigating some factors. Moreover, the UTAUT2 model has not yet received sufficient attention from researchers regarding the impact of data confidentiality and privacy concerns on e-PHR adoption, necessitating further investigation to bridge this gap. Accordingly, we have adapted the UTAUT2 model with four constructs that influence behavioral intention, namely, performance expectancy (PE), social influence (SI), effort expectancy (EE), and facilitating conditions (FC), that exert a significant pressure on the users' behavioral intention when making behavioral decisions. Furthermore, this study was conducted in response to the following three main questions:

Q1: How do confidentiality concerns impact patients' behavioral intention to adopt e-PHRs?
Q2: How do privacy concerns impact patients' behavioral intention to adopt e-PHRs?
Q3: What are the roles of confidentiality, privacy concerns, and some UTAUT2 variables, as mediation factors, in the adoption of e-PHRs?
The proposed research model is presented in Figure 1. The perspective constructs and their suggested hypothesis are discussed in the following sections.

### Performance expectancy

Performance expectancy (PE) refers to the user's judgment or expectancy that adopting digital technology is useful to improve their health. Many studies have found that performance expectancy can directly and significantly affect users' intention to use digital technology like e-PHRs (35, 40–42). The UTAUT model proposed and confirmed that performance expectancy positively affects individuals' behavioral intentions to adopt digital technology (36). However, a study conducted in Australia contradicts this and showed that the intention to use cloud-based mHealth services among patients was not influenced by performance expectancy (43). Thus, this study proposes the following hypotheses:

H1: Performance expectancy has a positive impact on users’ intention to adopt e-PHRs.
H2: Performance expectancy mediates the relationship between confidentiality and behavioral intention to adopt e-PHRs.
H3: Performance expectancy mediates the relationship between privacy and behavioral intention to adopt e-PHRs.

### Effort expectancy

Effort expectancy (EE) refers to the expected effort level users believe using a specific technology will require (35). Studies have identified that effort expectancy positively affects users' behavioral intention to adopt new technologies (44, 45). Regarding e-PHR services, the simpler that users perceive the use or operation of e-PHR services is, i.e., requiring less effort (including time and energy), the stronger their intention to adopt e-PHR services will be. Thus, this study proposes the following hypotheses:

H4: Effort expectancy has a positive impact on users' intention to adopt e-PHRs.
H5: Effort expectancy mediates the association between confidentiality and behavioral intention to adopt e-PHRs.
H6: Effort expectancy mediates the association between privacy and behavioral intention to adopt e-PHRs.

### Social influence

Social influence (SI) refers to the degree to which important people, such as family, friends, or experts, influence or are perceived to influence a person's decision to use e-PHRs. Multiple studies have argued that subjective norms are a key predictor in explaining and predicting a user's behavioral intention to adopt e-PHRs in different domains (40, 46–49). Research has shown that when important individuals recommend adopting e-PHRs, the patients’ trust in the products or services tends to increase significantly (50). However, some studies have found contradictory results, showing no significant effect on the intention to adopt digital health technologies like e-PHRs (51). For example, a study conducted in Australia on cloud-based mHealth services among patients and a study in Asia on Internet of Things (IoT) use found that the intention to adopt was not influenced by subjective norms, which are equivalent to social influence (43, 52). In this context, we propose the following hypotheses:

H7: Social influence has a positive impact on users' intention to adopt e-PHRs.
H8: Social influence mediates the association between privacy and behavioral intention to adopt e-PHRs.
H9: Social influence mediates the association between confidentiality and behavioral intention to adopt e-PHRs.

### Facilitating conditions

Facilitating conditions (FC) refer to how accessible a user perceives a resource that assists in emerging technology adoption to be (53). Studies have shown that facilitating conditions significantly influence a user's behavioral intention to adopt information technology like e-PHRs (54, 55). However, multiple studies have found that facilitating conditions did not affect behavioral intention to adopt information technology. Studies conducted in the USA, South Africa, and Asia showed that facilitating conditions were not significantly associated with behavioral intention to adopt or use health-related information technology in different domains. However, in this study, the aim was to assess the effect of facilitating conditions, with the following hypotheses:

H10: Facilitating conditions have a positive effect on users’ behavioral intention on the adoption of e-PHRs.
H11: Facilitating conditions mediate the association between privacy and behavioral intention to adopt e-PHRs.
H12: Facilitating conditions mediate the association between confidentiality and behavioral intention to adopt e-PHRs.

### Privacy concerns

Privacy concerns refer to the degree to which users are concerned about the disclosure of personal health information. In the context of utilizing digital technology, a user who is unaware of or unfamiliar with the service is more likely to be highly concerned about personal information abuse or leakage. This is especially true if their information is used for other purposes without their consent, leading to personal information leakage and loss of privacy. If users believe that there are privacy issues involved in using e-PHRs, they may reject suggestions from others and react negatively to adopting e-PHRs (56, 57). Research studies have identified that when one thinks that there might be a privacy concern in using digital technology, one may reject or reduce one’s intention to adopt the technology (55, 58). Privacy concerns also affect facilitating conditions and effort expectancy (59). Thus, the authors of this study propose the following hypotheses:

H13: Users' privacy issues significantly influence effort expectancy.
H14: Users' privacy issues significantly influence performance expectancy.
H15: Users' privacy issues significantly influence intention to adopt e-PHRs.
H16: Users' privacy issues significantly influence social influence.
H17: Users' privacy issues significantly influence the facilitating conditions.

### Confidentiality concerns

Confidentiality involves restricting unauthorized individuals' access to personal information and keeping communication confidential (60). However, confidentiality is becoming a major problem in the adoption of digital technology in the healthcare system. Various studies have investigated the effect of confidentiality concerns when utilizing information technology in various domains (61, 62). According to a study conducted among patients to assess the effect of confidentiality concerns on the adoption of e-PHRs, confidentiality concerns positively affect perceived usefulness and perceived ease of use, which in turn influences users’ intention to adopt e-PHRs (29). The authors of this study propose the following hypotheses.

H18: Personal health data confidentiality has a significant effect on effort expectancy.
H19: Personal health data confidentiality has a significant effect on performance expectancy.
H20: Personal health data confidentiality has a significant effect on social influence.
H21: Personal health data confidentiality has a significant effect on facilitating the condition.
H22: Personal health data confidentiality has a significant impact on intention to adopt e-PHRs.

## Method

### Study design and setting

An institution-based cross-sectional study was used to assess the impact of confidentiality and privacy concerns on behavioral intention to adopt e-PHRs among chronic patients in the Iluu Aba Bora Zone in southwest Ethiopia from 24 July to 17 September 2023.

### Study participants and sampling size determination

Patients with chronic diseases, including hypertension, diabetes mellitus, chronic cardiac failure, HIV/AIDS, chronic respiratory disease, and asthma, who had follow-ups in healthcare facilities in the Iluu Aba Bora Zone were the source population. The patients who had follow-ups in healthcare facilities in the Illu Aba Bora Zone and were available during data collection were considered the study population. Chronic patients younger than 18 years old and those unable to respond due to a significant illness were not included in the study. Based on the assumption of structural equation modeling (SEM), the sample size for this study was computed using a 1:10 ratio based on the number of free parameters in the theoretical model. As a result, after taking a free parameter ratio of 10, a non-response rate of 10%, and 71 parameters into account, the final sample size was 781.

### Sampling procedures

A systematic random sampling technique was employed to select the study participants. The selected patients were interviewed using a structured questionnaire in the local language. During the data collection period, the interval size (*K*) per month was calculated using the formula *K* = N/n, where *K* denotes the interval size, *N* denotes the total average number of selected chronic patients who visit the healthcare facilities in the Illu Aba Bora Zone per month (976), and *n*  denotes the number of chronic patients (781). Thus, *K* = 1.2, which meant that we selected every second patient was selected by considering their setting position in a list of follow-up appointments. An integer (2) was then randomly selected through the lottery method between 1 and *k* (2), and every *k*th (2) record was selected.

### Data collection tools and procedures and data quality control

A standard interviewer-administered questionnaire, adapted from the original instrument developed by Davis's and Venkatesh's study using the modified Technology Acceptance Model (TAM) (29) and UTAUT2 model (29, 35, 40, 63), was administered. Sociodemographic information, UTAUT2 components [PE, EE, FC, SI, and behavioral intention to adopt a personal health record system (BIAPHRS)], and additional factors of confidentiality and privacy concerns were all included in the questionnaire by referencing previously published articles. Hence, the questionnaire was modified according to the objectives of this study. A five-point Likert scale was used to evaluate the constructs, with “1” indicating “strongly disagree” and “5” indicating “strongly agree.” The questionnaire was initially developed in the English language and translated into the local language by experts. The questionnaire in the Afan Oromo language was re-translated back to English to check the consistency of the translation. Moreover, 10% of the sample underwent a pretest at Jimma University Specialized Referral Hospital. Based on the results of the pretest, the tool was revised. Three BSc health informatics professionals and four MPH health professionals were assigned to the data collection and supervision, respectively. The study's objective, data collection method, questionnaire content, and ethical concerns were covered in a training session for the data collectors and supervisors.

### Data processing and analysis

The data were entered into EpiData version 4.1 and further exported to SPSS version 26 for data cleaning and coding before the data analysis was conducted. Descriptive statistics were employed to explore the sociodemographic characteristics of the study participants and their confidentiality concerns, privacy concerns, and behavioral intentions to adopt e-PHRs. Model constructs were assessed using SEM in the Analysis of Moment Structure (AMOS) version 23 software. Reliability and validity tests were employed to assess the degree to which a variable was consistent and how effectively the selected construct items measured the construct. Since not all the indicators were equally reliable, construct reliability was evaluated using a composite reliability exceeding 0.7. In the SEM analysis, weighted composite reliability was more accurate than unweighted Cronbach's alpha in assessing the construct reliability. An average variance extracted (AVE) value ≥ 0.5, a factor loading value of ≥0.6 for each construct, and composite reliability were used to assess the convergent validity. The Fornell–Larcker criterion was used to assess the discriminant validity. It was supported if a construct's square root of AVE for each latent variable be greater than the correlation between that variable and any other latent variable in the model.

The Mahalanobis distance was used to check the assumption of a multivariate outlier. Furthermore, multivariate kurtosis <5 and a critical ratio (CR) between −1.96 and +1.96 were used to assess the normality. A variance inflation factor (VIF) value < 10, tolerance >0.1, and correlation coefficient <0.8 between exogenous constructs were employed to assess multicollinearity. Moreover, the correlation between exogenous constructs is less than 0.8. We applied the maximum log likelihood method to estimate the measurement and structural models. The test measurement model was subjected to confirmatory factor analysis (CFA) with standardized values, which illustrated how the measured variables combined to represent constructs. As part of the confirmatory factor analysis, the correlation between constructs and factor loadings for each item was evaluated, and the factor loading value for each item was greater than 0.5 (64, 65). A chi-square ratio < 3, comparative fit index (CFI) > 0.9, goodness-of-fit index (GFI) > 0.9, adjusted goodness-of-fit index (AGFI) > 0.8, root-mean-square deviation approximation (RMSDA) < 0.08, and root-mean-square of standardized residual (RMSR) < 0.08 were used to assess the goodness of fit of the model. To assess the relationship between the exogenous and endogenous variables, the critical ratio, the path coefficient, and the squared multiple correlation coefficient (*R*^2^) were estimated. Furthermore, 95% CIs and a *p*-value < 0.05 were used to assess statistical significance.

## Result

### Sociodemographic characteristics of chronic patients

A total of 680 chronic patients were interviewed, with a response rate of 87.3%. The study participants' mean age was 43.9 years (SD ± 12.9 years). The majority of the study participants were urban residents. Regarding educational status, most of the study participants had informal education, and most of the study participants were married. Nearly half of the study participants were Orthodox, followed by Muslims (Table 1).

**Table 1:** Sociodemographic characteristics of the chronic patients in the Illu Aba Bora Zone, southwest Ethiopia, in 2023.

Variable	Category	Frequency (*n*)	%
Age	43.9 ± 12.9		
Gender	Female	367	54.0
	Male	313	46.0
Residence	Urban	545	80.1
	Rural	135	19.9
Educational status	Non-formal education	242	35.5
	Primary education	114	16.8
	Secondary education	93	13.7
	Higher education	231	34.0
Marital status	Single	118	17.4
	Married	453	66.6
	Divorced	62	9.1
	Widowed	47	6.9
Religion	Orthodox	321	47.2
	Muslim	254	37.4
	Others	105	15.4

### Behavioral intention to adopt e-PHRs

The study findings suggest that 288 (42.4%; 95% CI: 39.0, 46.2) chronic patients intended to adopt e-PHRs. The mean score of behavioral intention to adopt e-PHRs was 3.999 (SD ± 1.001) among the chronic patients in Illu Aba Bora Zone, southwest Ethiopia.

### Measurement model

**Figure 2:**
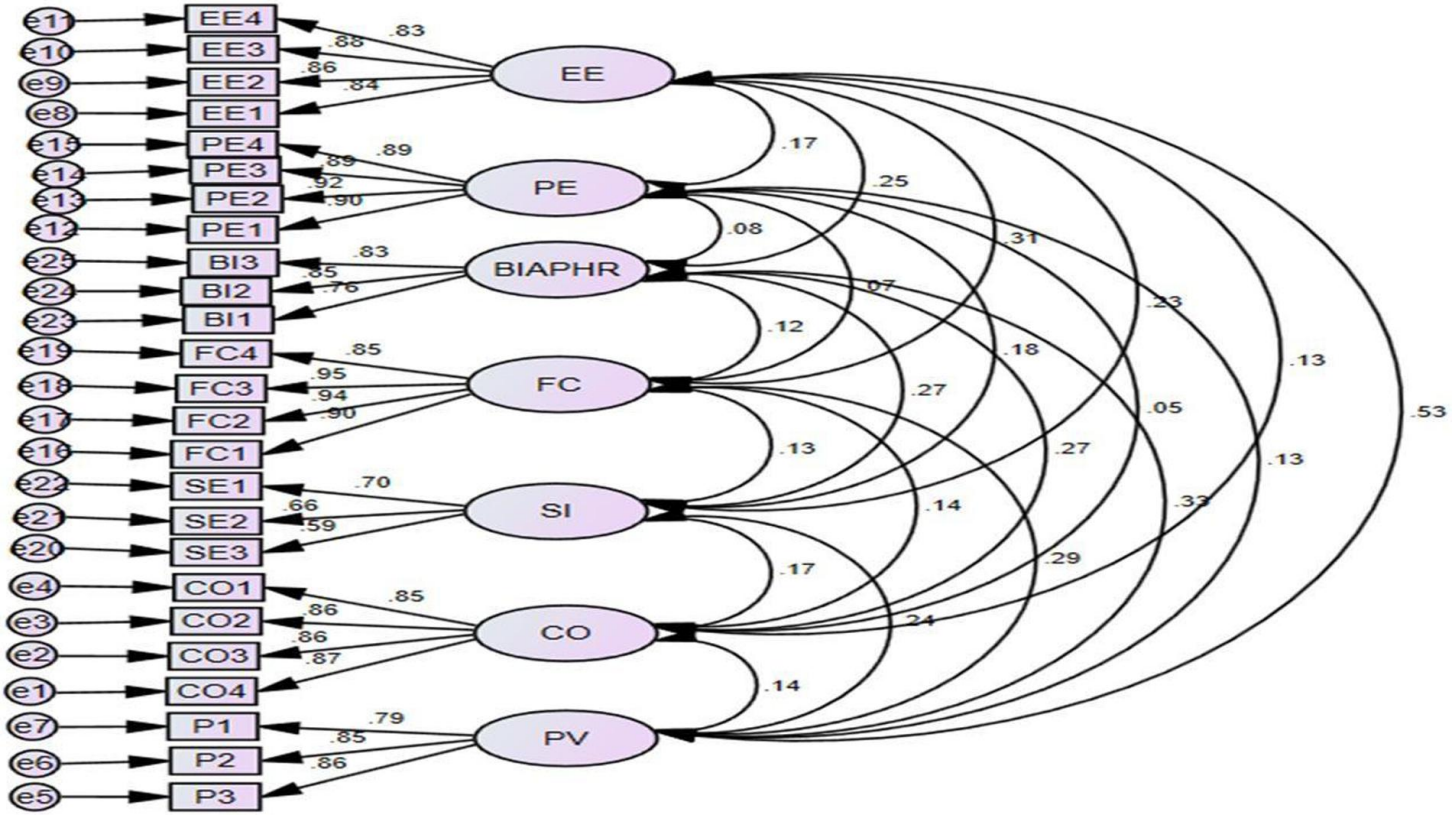
CFA of the behavioral intention to adopt e-PHRs among chronic patients in Illu Aba Bora, southwest Ethiopia, in 2023.

We assessed the measurement model by calculating the model fitness, internal consistency, convergent validity, and discriminant validity indicators/items using CFA, as shown in Figure 2.

### Reliability and validity test

The correlations between the constructs are presented in Table 2. Using the Fornell–Larcker criterion, the square root of the AVE of all the constructs was significantly larger than the correlation coefficient between the other constructs, indicating that the measurement model had satisfactory discriminant validity. The results of this study indicated that the multivariate kurtosis value exceeded 5 (kurtosis = 103.8), and the multivariate critical ratio fell outside the range of −1.69 to +1.69 (CR = 54.2). In this scenario, the non-parametric bootstrapping methods assist with analyzing non-normally distributed data by resampling it under the assumption of a normal distribution. This approach helps estimate the significance of the path coefficients, standard errors, and confidence intervals. Thus, a bootstrap with 5,000 resampling iterations was conducted to obtain the maximum consistency possible in the results for structural model path significance with a 95% CI bias-corrected confidence interval.

**Table 2:** Convergent validity between constructs in predicting the impact of confidentiality and privacy concerns on the behavioral intention to adopt e-PHRs among chronic patients in southwest Ethiopia in 2023.

Constructs	Item	Standard loading	Average variance extracted (AVE)	Composite reliability
PE	PE1	0.90	0.814	0.946
	PE2	0.92		
	PE3	0.89		
	PE4	0.89		
EE	EE1	0.84	0.726	0.914
	EE2	0.86		
	EE3	0.88		
	EE4	0.83		
FC	FC1	0.90	0.827	0.950
	FC2	0.94		
	FC3	0.95		
	FC4	0.85		
SI	SI1	0.59	0.512	0.71
	SI2	0.66		
	SI3	0.70		
CO	CO1	0.85	0.738	0.919
	CO2	0.86		
	CO3	0.86		
	CO4	0.87		
PV	PV1	0.79	0.699	0.874
	PV2	0.85		
	PV3	0.86		
BIAPHR	BIAPHR1	0.76	0.663	0.855
	BIAPHR2	0.85		
	BIAPHR3	0.83		

CO, confidentiality; PV, privacy; PE, performance expectancy; FC, facilitating conditions; EE, effort expectancy; SI, social influence; BIAPHR, behavioral intention to adopt personal health record.

### Goodness of fit

We evaluated the model fit indices using confirmatory factor analysis in the SEM model by comparing them to their respective threshold values. If the results met or exceeded these thresholds, we concluded that the model fit indices were acceptable. We calculated the following values: *Χ*^2^ difference = 3.0, minimum discrepancy of confirmatory factor analysis/degrees of freedom (CMIN/DF) = 2.79, GFI = 0.92, AGFI = 0.90, CFI = 0.96, tucker-lewis index (TLI) = 0.96, PClose = 0.29, root mean square error approximation (RMSEA) = 0.05, and standardized root mean squared residual (SRMR) = 0.05. Therefore, the goodness-of-fit indices were acceptable in this study.

Table 2 presents the convergent validity between the constructs that affect behavioral intention to adopt e-PHRs. The composite reliability ranged from 0.71 to 0.946, which shows that the suggested model's construct reliability was achieved. The standard loading coefficients of all factors ranged from 0.59 to 0.95 and were greater than 0.5, which suggests that the convergent validity requirement was achieved. Furthermore, the AVE value was greater than 0.5, indicating the suggested construct reliability was achieved. From the above indicators, it can be concluded that the questionnaire met the requirements of composite reliability and had good convergent validity.

The correlations between the constructs are presented in Table 3. Using the Fornell–Larcker criterion, the square root of the AVE of all the constructs was significantly larger than the correlation coefficient between the other constructs, indicating that the measurement model had satisfactory discriminant validity.

**Table 3:** Discriminant validity between constructs in predicting behavioral intention to adopt e-PHRs among chronic patients in southwest Ethiopia in 2023.

Construct	CO	PV	PE	FC	EE	SI	BIAPHR
CO	**0** **.** **859**						
PV	0.141	**0**.**836**					
PE	0.051	0.127	**0**.**902**				
FC	0.140	0.289	0.066	**0**.**910**			
EE	0.133	0.529	0.000	0.000	**0**.**852**		
SI	0.166	0.245	0.177	0.134	0.231	**0**.**651**	
BIAPHR	0.266	0.335	0.084	0.121	0.250	0.269	**0.814**

CO, confidentiality; PV, privacy; PE, performance expectancy; FC, facilitating condition; EE, effort expectancy; SI, social influence; BIAPHR, BEHAVIORAL intention to adopt personal health record.

Bold value indicates discriminant validity between constructs.

### Structural equation modeling

Our UTAUT2 model consisted of confidentiality, privacy, performance expectancy, effort expectancy, facilitating conditions, and social influences and their effects on behavioral intention to adopt e-PHRs. The results showed that SI had a significant effect on behavioral intention to adopt e-PHRs (*β* = 0.157, 95% CI: 0.280, 0.475, *p* < 0.01). Privacy was found to be a factor that affects behavioral intention to adopt e-PHRs (*β* = 0.216, 95% CI: 0.001, 0.207, *p* < 0.05). Importantly, confidentiality was found to be a factor that affects behavioral intention to adopt e-PHRs (*β* = 0.156, 95% CI: 0.101, 0.306, *p* < 0.01).

Furthermore, privacy concerns had a significant effect on chronic patients’ EE, SI, FC, and PE and their behavioral intention to adopt e-PHRs (*β* = 0.517, 95% CI: 0.425, 0.630, *p* < 0.05; *β* = 0.227, 95% CI: 0.201, 0.410, *p* < 0.05; *β* = 0.349, 95% CI: 0.009, 0.162, *p* < 0.05; *β* = 0.087, 95% CI: 0.009, 0.162, *p* < 0.05; *β* = 0.087, 95% CI: 0.233, 0.470, *p* < 0.05; *β* = 0.180, 95% CI: 0.057, 0.32, *p* < 0.05, respectively). Confidentiality concerns regarding personal health information had a significant effect on the patients’ SI and FC and their behavioral intention to adopt e-PHRs (*β* = 0.108, 95% CI: 0.026, 0.201, *p* < 0.05; *β* = 0.103, 95% CI: 0.026, 0.189, *p* < 0.05). Table 4 illustrates the structural equation modeling that shows how each predictor/item affects behavioral intention to adopt e-PHRs.

**Table 4:** SEM analysis of chronic patients’ behavioral intention to adopt e-PHRs in southwest Ethiopia in 2023.

Path	Hypothesis	*Β*	SE	CR	*p*-Value	LCI	UCI	Description
PE→BIAPHR	H1	0.007	0.028	0.238	0.812	-0.042	0.061	Not supported
EE→BIAPHR	H2	0.064	0.047	1.366	0.172	-0.028	0.162	Not supported
SI→BIAPHR	H3	0.157	0.049	3.235	0.001	0.053	0.281	Supported
FC→BIAPHR	H4	0.015	0.032	0.456	0.648	-0.078	0.056	Not supported
PV→EE	H5	0.517	0.041	12.697	0.000	0.425	0.630	Supported
PV→PE	H6	0.180	0.056	3.225	0.001	0.057	0.320	Supported
PV→BIAPHR	H7	0.216	0.051	4.230	0.000	0.101	0.347	Supported
PV→SI	H8	0.227	0.047	4.784	0.000	0.201	0.410	Supported
PV→FC	H9	0.349	0.050	7.044	0.000	0.233	0.470	Supported
CO→EE	H10	0.050	0.031	1.620	0.105	-0.008	0.115	Not supported
CO→PE	H11	0.038	0.047	0.810	0.418	-0.056	0.140	Not supported
CO→SI	H12	0.108	0.039	2.791	0.005	0.026	0.201	Supported
CO→FC	H13	0.103	0.041	2.536	0.011	0.026	0.189	Supported
CO→BIAPHR	H14	0.156	0.033	4.718	0.000	0.081	0.241	Supported

**Figure 3:**
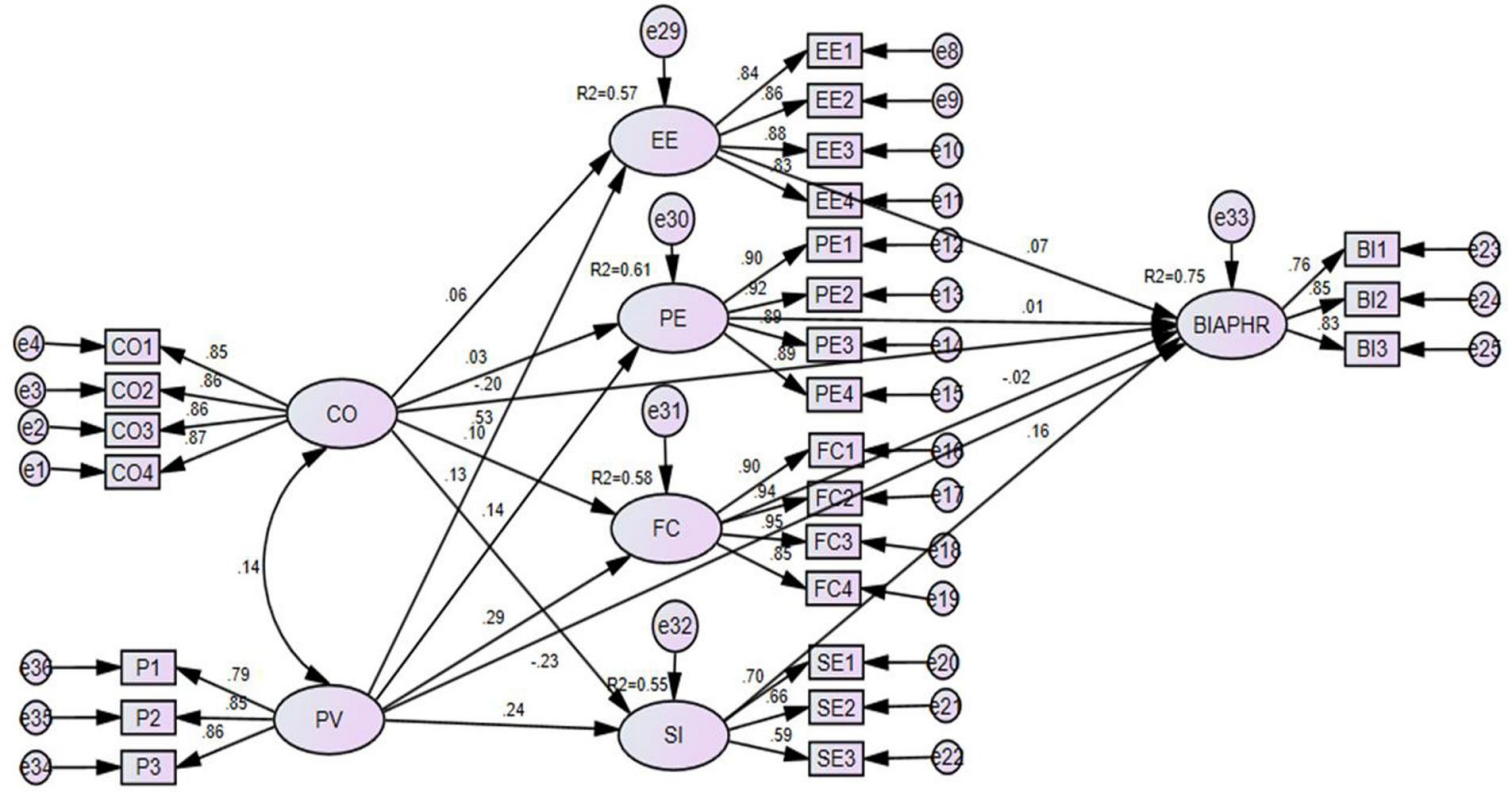
SEM analysis of the impact of confidentiality and privacy concerns on the behavioral intention to adopt e-PHRs among chronic patients in southwest Ethiopia in 2023. CO, confidentiality; PV, privacy; EE, effort expectancy; PE, perceived expectancy; FC, facilitating conditions; SI, social influence; BIAPHR, behavioral intention to adopt personal health record.

Chronic patients' behavioral intention to adopt e-PHRs was influenced the most by data privacy concerns, followed by social influence and data confidentiality concerns, and these factors played a significant role in the chronic patients’ behavioral intention to adopt e-PHRs. Furthermore, confidentiality, privacy, performance expectancy, effort expectancy, facilitating conditions, and social influence accounted for 75% of the variance (*R*^2^) in the behavioral intention to adopt e-PHRs among the chronic patients. Confidentiality and privacy accounted for 57% of the variance (*R*^2^) in effort expectancy, 61% in performance expectancy, 58% in facilitating conditions, and 55% in social influence. A more detailed illustration of the data analysis is provided in Figure 3.

### Mediation effect

Eight alternative mediation paths were analyzed based on their impact and significance in predicting the behavioral intention of chronic patients to adopt e-PHRs. Partial mediation occurs when a construct's direct, indirect, and total effects are all statistically significant; full mediation occurs when the direct and indirect effects are significant but the total impact is insignificant or negligible. To verify the mediation in our analysis, we set a threshold for a meaningful indirect effect of a *p*-value of 0.05 or less.

As shown in Table 5, the relationship between confidentiality and the patients’ behavioral intention to adopt an e-PHRS was mediated by social influence and facilitating conditions, with a *p*-value of less than 0.001. Social influence and confidentiality were found to be statistically significant in influencing the patients’ behavioral intention to adopt the e-PHRs.

**Table 5:** Mediating effects of PE, EE, SI, and FC on the adoption of e-PHRs among chronic patients in southwest Ethiopia in 2023.

Constructs	Hypothesis	Effect	*Β*	*p*-Value	Result	Decision
CO→PE→BIAPHR	H2	Total	0.033	0.433	Direct mediation	Not supported
		Direct	0.033	0.433		
		Indirect	0.000	0.000***		
PV→PE→BIAPHR	H3	Total	0.136	0.06	Direct mediation	Not supported
		Direct	0.136	0.06		
		Indirect	0.000	0.000***		
CO→EE→BIAPHR	H5	Total	0.060	0.13	Full mediation	Not supported
		Direct	0.060	0.012*		
		Indirect	0.000	0.000***		
PV→EE→BIAPHR	H6	Total	0.531	0.093	Direct mediation	Not supported
		Direct	0.531	0.093		
		Indirect	0.000	0.000***		
PV→ SI→BIAPHR	H8	Total	0.239	0.000***	Partial mediation	Supported
		Direct	0.239	0.000***		
		Indirect	0.000	0.000***		
CO→ SI→BIAPHR	H9	Total	0.134	0.008**	Partial mediation	Supported
		Direct	0.134	0.008**		
		Indirect	0.000	0.000***		
PV→FC→BIAPHR	H11	Total	0.29	0.02*	Direct mediation	Not supported
		Direct	0.290	0.13		
		Indirect	0.000	0.001**		
CO→FC→BIAPHR	H12	Total	0.101	0.01**	Partial mediation	Supported
		Direct	0.101	0.01**		
		Indirect	0.000	0.000***		

* indicates significance at *p*-value < 0.05, ** indicates significance at *p*-value < 0.01, and *** indicates significance at *p*-value < 0.001.

However, facilitating conditions did not statistically significantly influence the chronic patients' behavioral intention to adopt e-PHRs. In addition, the relationship between privacy and the chronic patients' behavioral intention to adopt e-PHRs was mediated by social influence, with a significant *p*-value of less than 0.001.

## Discussion

Policymakers, patients, and healthcare professionals have stated that adopting e-PHRs benefits healthcare organizations. Furthermore, patients are the primary beneficiaries, using e-PHRs to access a wide range of credible health-related information. As the adoption of e-PHRs is influenced by confidentiality and privacy concerns among chronic patients, this study examined the impact of these concerns on patients’ behavioral intention to adopt e-PHRs in the Iluu Aba Bora Zone, southwest Ethiopia. In addition, the study investigated the chronic patients’ behavioral intention to adopt e-PHRs. The results showed that 288 chronic patients (42.6%) (95% CI: 39.0, 46.2) expressed an intention to adopt e-PHRs to manage, promote, and improve their health.

This study’s findings are lower than those of studies conducted in the USA (66) and Canada (67), which focused on assessing patients’ behavioral intentions toward some wearable devices. This discrepancy may be attributed to the lower internet penetration rate in Ethiopia (16.7%) and the limited advancement of digital health technology in the healthcare system. Another possible explanation for this discrepancy may be related to variations in knowledge and attitude differences toward obtaining health-related information from digital technology in developing countries. Furthermore, the difference in sample sizes could be another contributing factor. The studies conducted in Canada and the USA used much larger sample sizes (*n* = 4,109 and *n* = 4,551, respectively) compared to our study (*n* = 680). Our study’s findings are somewhat similar to studies conducted in Ethiopia among patients with diabetes mellitus (47.1%) (65) and chronic patients (46.7%) (21). This may be due to the small difference in sample sizes and the comparable sociodemographic characteristics of the study participants. However, the findings of this study were lower than another study conducted in southwest Ethiopia among healthcare professionals, which reported 57.6% (22). This discrepancy may be due to differences in the use of digital health technology between healthcare professionals and chronic patients. Another possible explanation for this difference could be the lack of digital health literacy among the study participants.

This study found that the behavioral intention to adopt e-PHRs was significantly linked to social influence, privacy, and confidentiality. Conversely, performance expectancy, effort expectancy, and facilitating conditions were not significantly associated with the patients’ behavioral intention to adopt e-PHRs. Thus, hypotheses H7, H15, and H22 were supported by this study's findings. The results indicate that behavioral intention to use e-PHRs among chronic patients is affected by privacy and confidentiality, both directly and indirectly. In addition, social influence had a direct impact on the patients’ behavioral intention to adopt e-PHRs (*β* = 0.157, 95% CI: 0.280, 0.475, *p* < 0.01).

This finding underscores the importance of social support in the adoption of e-PHRs among chronic patients. Specifically, those who have a motivating individual in their lives are more likely to embrace e-PHRs, particularly when these technologies promise to enhance their health management and provide easy access to their health information. In addition, the influence of peer opinions plays a significant role in the decision-making process when chronic patients are considering new technology adoption. This conclusion aligns with previous research conducted in Saudi Arabia (49). Interestingly, this study revealed that concerns about confidentiality had a direct impact on the patients’ behavioral intention to adopt e-PHRs (*β* = 0.156, 95% CI: 0.101, 0.306, *p* < 0.01), thereby supporting hypothesis H22. Technologies that prioritize and ensure the confidentiality of personal information are more likely to be adopted by users. This finding is consistent with previous studies conducted across various countries and among diverse populations (29). Moreover, this study identified health information privacy concerns as another critical factor influencing the behavioral intention to adopt e-PHRs among chronic patients. The analysis revealed a direct and indirect effect on this intention (*β* = 0.216, 95% CI: 0.001, 0.207, *p* < 0.05), supporting hypothesis H15. Participants who believe that e-PHRs can safeguard their private medical information from unauthorized access are significantly more inclined to adopt this technology (29). These findings are reinforced by a previous study conducted in various healthcare settings, which examined the impact of privacy concerns on the implementation of new technologies aimed at improving health outcomes (58). Overall, this study highlights the multifaceted nature of technology adoption among chronic patients, emphasizing the critical roles of social support, confidentiality, and privacy assurance in facilitating the use of e-PHRs.

## Conclusion and limitations of the study

This study assessed the impact of privacy and confidentiality concerns on chronic patients’ behavioral intention to adopt e-PHRs in Iluu Aba Bora, southwest Ethiopia, by extending the UTAUT2 model, and enriches the literature on the impact of privacy and confidentiality concerns on patients’ behavioral intention to adopt e-PHRs. A better understanding of the factors that affect patients’ behavioral intentions to adopt e-PHRs will help the government and policymakers to implement the correct strategies for the diffusion of the system in the country. The findings of this study illustrate the significance of privacy and confidentiality concerns in determining chronic patients’ behavioral intention to adopt e-PHRs. The mediation effects of these factors were partially confirmed in this study. Only the mediation role of facilitating conditions and social influence in the relationship between confidentiality, privacy, and behavioral intention was confirmed in this study. The developed model explained 75.0% of the variance in the patients’ behavioral intention to adopt e-PHRs. Consequently, future studies should consider adding other context-related factors such as e-health literacy, health status, trust, and patients' self-efficacy, as these may affect the adoption of e-PHRs. Even though this study provides empirical evidence, it has some limitations that should be considered. This study only focused on some concerns and limited factors to assess their effect on the adoption of e-PHRs. Furthermore, this was a cross-sectional quantitative study, and it only assessed the correlation between variables, not causality. Furthermore, it is important to note that this study did not include elderly participants, resulting in a sample predominantly composed of younger individuals aged between 20 and 60 years. This demographic limitation may impact the generalizability of the findings to older populations.

## Data Availability

The data analyzed in this study are subject to the following licenses/restrictions: as the data are sensitive, we could not upload the data, but we can share it upon reasonable request. Requests to access these datasets should be directed to Sisay Yitayih Kassie, sishaimanot@gmail.com.
